# Primary hepatic malignant triton tumor mimicking hepatocellular carcinoma by demonstrating arterial-phase hypervascularity and subsequent washout on dynamic contrast-enhanced imaging: a case report and literature review

**DOI:** 10.3389/fmed.2024.1361690

**Published:** 2024-03-04

**Authors:** Bo Zhou, Canyang Zhan, Yang Tian, Zhenzhen Gao, Sheng Yan

**Affiliations:** ^1^Department of Hepatobiliary and Pancreatic Surgery, The Second Affiliated Hospital, Zhejiang University School of Medicine, Hangzhou, China; ^2^Department of Neonatology, Children’s Hospital, School of Medicine, Zhejiang University, Hangzhou, China

**Keywords:** hepatic malignant triton tumor, hepatocellular carcinoma, complete surgical removal, prognosis, case report

## Abstract

**Background:**

Malignant Triton tumor (MTT) is a relatively rare subtype of malignant peripheral nerve sheath tumor (MPNST) characterized by rhabdomyosarcoma differentiation. There are no distinct features of MTT, and it is easy to misdiagnose preoperatively.

**Case presentation:**

Here, we describe a rare case of primary hepatic MTT in a 56-year-old male who presented with nonspecific abdominal pain for 1 day. Magnetic resonance imaging and abdominal computed tomography revealed an extremely large mass located in the right liver with intratumoral hemorrhage, arterial-phase hypervascularity and subsequent washout on dynamic contrast-enhanced imaging and the possibility of intrahepatic metastasis. Tumor marker levels revealed only an elevated level of alpha-fetoprotein (AFP: 5304.0 ng/mL). Then, he received transcatheter arterial chemoembolization combined with lenvatinib and pembrolizumab, and he was diagnosed with hepatocellular carcinoma. After 3 months of neoadjuvant therapy, we resected the hepatic cancer and adherent diaphragmatic pleura. MTT was confirmed by postoperative pathology and immunohistochemistry.

**Conclusion:**

Despite the preoperative diagnosis of hepatocellular carcinoma with a rising serum AFP level, typical CT and MRI findings, histopathology assessment showing MPNST with rhabdomyosarcoma differentiation confirms the diagnosis of primary hepatic MTT.

## Introduction

Malignant Triton tumor (MTT), originally described by Masson and Martin in 1932, is an aggressive and frequent subtype of malignant peripheral nerve sheath tumor (MPNST) characterized by rhabdomyosarcoma (1). The most common site of MTT is the neck, head and trunk, but it has also been observed infrequently in the mediastinum, lung, abdomen, and prostate tissue (2–4). In 50% of cases, MTT is associated with neurofibromatosis type 1 (NF-1) and has an equal sex distribution (5, 6). Due to their size and location, these tumors are difficult to resect completely and have a poor prognosis because they are highly malignant and frequently recur (7).

Herein, we report a rare case of primary hepatic MTT without NF-1, mimicking hepatocellular carcinoma (HCC), which was successfully and radically surgically excised.

## Case presentation

A 56-year-old man without a history of NF-1 was admitted to our hospital because of nonspecific abdominal pain for 1 day and a history of hepatitis B without treatment. The detection value of hepatitis B virus (HBV) DNA was 14,400 IU/mL. Tumor marker levels revealed only an elevated level of alpha-fetoprotein (AFP: 5304.0 ng/mL). Liver dysfunctions, including AST 134 U/L and ALT 116 U/L, were detected. Other laboratory findings, including serum creatinine levels, leukocyte and platelet counts, hemoglobin levels and blood coagulation, were within normal ranges.

**Figure 1:**
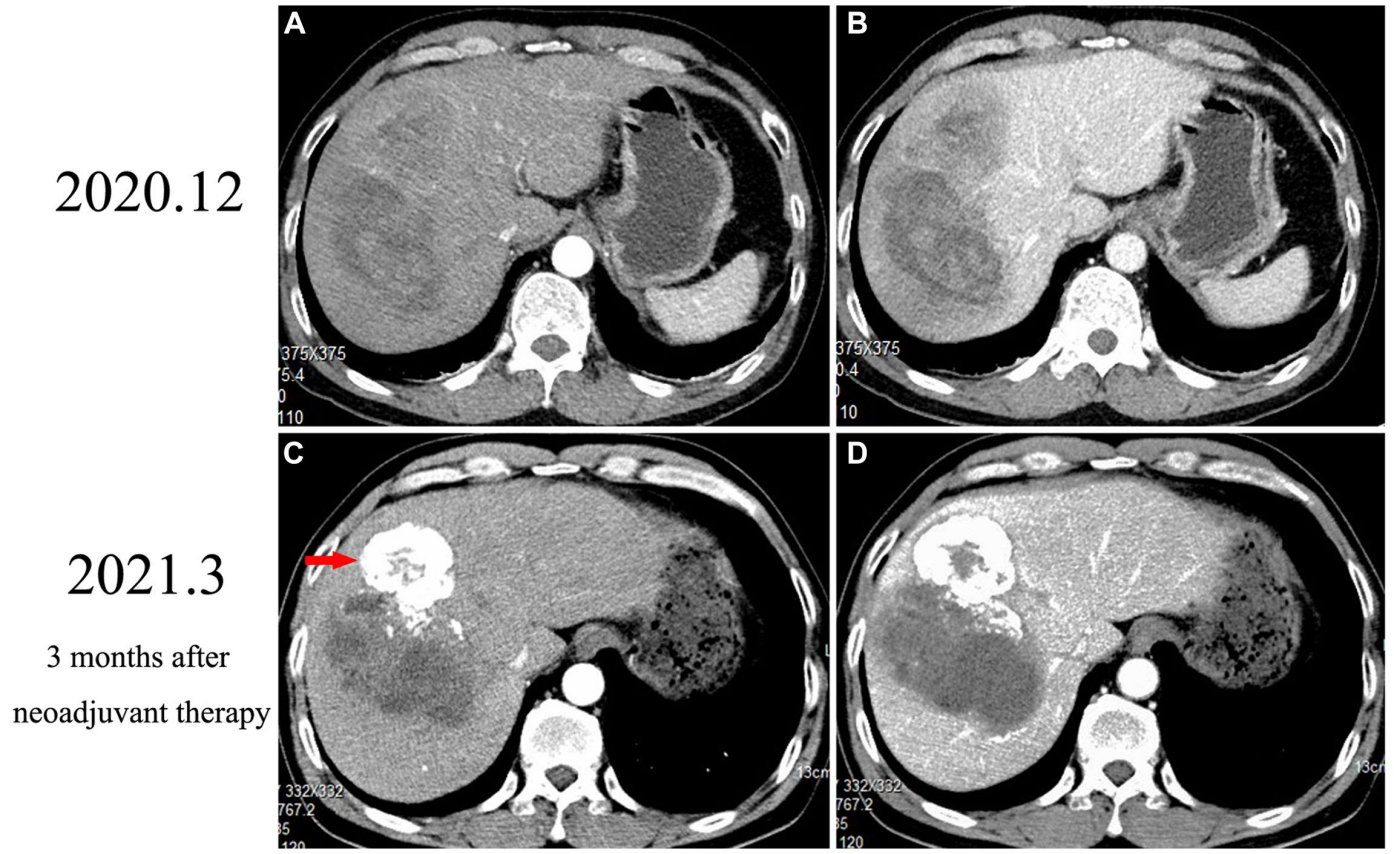
An abdominal computed tomography scan confirmed an extremely large mixed-density mass measuring approximately 13 * 10.1 * 9.2 cm located in the right liver with intratumoral hemorrhage. The tumor exhibited an enhancement pattern as fast-in and fast-out **(A,B)**. After 3 months of neoadjuvant therapy, most of the mass was necrotic, with iodine oil deposition (red arrow) and slight local enhancement **(C,D)**.

**Figure 2:**
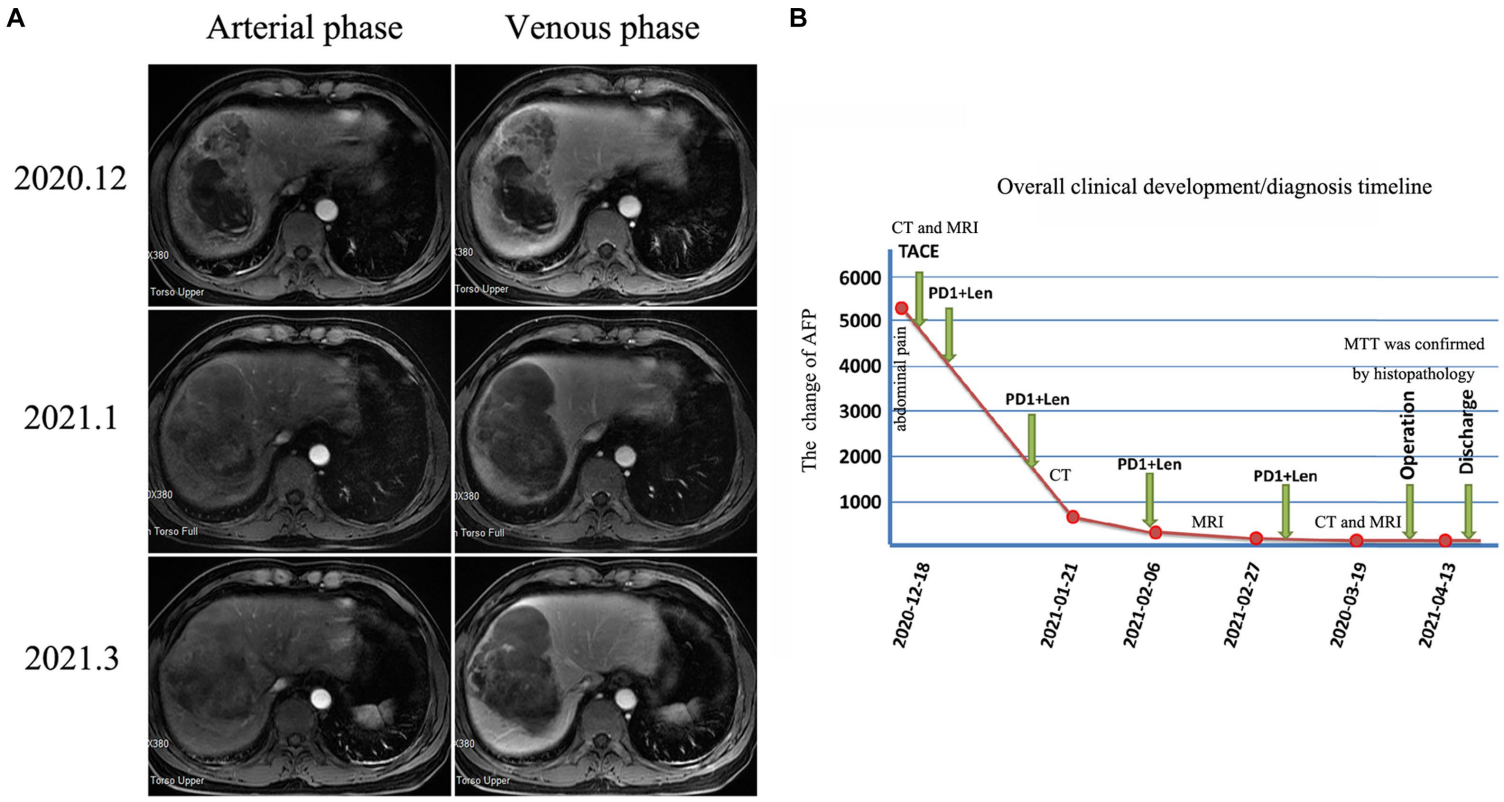
Changes in magnetic resonance imaging and AFP levels during patient treatment After 3 months of neoadjuvant therapy, MRI revealed that most of the lesion was necrotic, with slight local enhancement **(A)**. The overall clinical development/diagnosis timeline **(B)**.

Abdominal contrast-enhanced computed tomography (CT) revealed an extremely large mixed-density mass measuring approximately 13 * 10.1 * 9.2 cm and located in the right liver (mainly in segment VIII) with intratumoral hemorrhage invading the right anterior branch of the portal vein. An enhancement pattern, such as fast-in or fast-out, was also observed (Figures 1A,B). Furthermore, magnetic resonance (MRI) confirmed the presence of the lesion, which was hyperintense at T2 and had significant uneven enhancement (Figure 2A). Moreover, multiple abnormal small nodules were observed in the arterial and venous phases, indicating the possibility of intrahepatic metastasis. According to the NCCN Guidelines’ recommendations for diagnosis of HCC, a diagnosis of HCC can be made. When the diagnosis is established by imaging criteria, patients can be treated and biopsy confirmation is not needed. Because of the possibility of HCC with intrahepatic metastasis, the patient underwent transcatheter arterial chemoembolization (TACE) combined with 8 mg of lenvatinib orally once daily as well as intravenous pembrolizumab every 3 weeks. In addition, he received antiviral treatment (entecavir) for HBV. Then, he underwent close follow-up, which was performed an average of once a month. However, his AFP level significantly decreased. CT (Figures 1C,D) and MRI (after 3 months) revealed a large mass in the right lobe of the liver, with a length of approximately 120 * 83 * 82 mm. Most of the lesions were necrotic, with iodine oil deposition and slight local enhancement (Figure 2A). The level of AFP decreased to 5.9 ng/mL (Figure 2B). At 3 months, a partial response (PR) was observed according to the modified Response Evaluation Criteria in Solid Tumors (mRECIST; Figure 2A). After multidisciplinary discussion, the hepatic cancer and adherent part of the diaphragmatic pleura were resected. The patient recovered well after surgery, without any postoperative complications.

**Figure 3:**
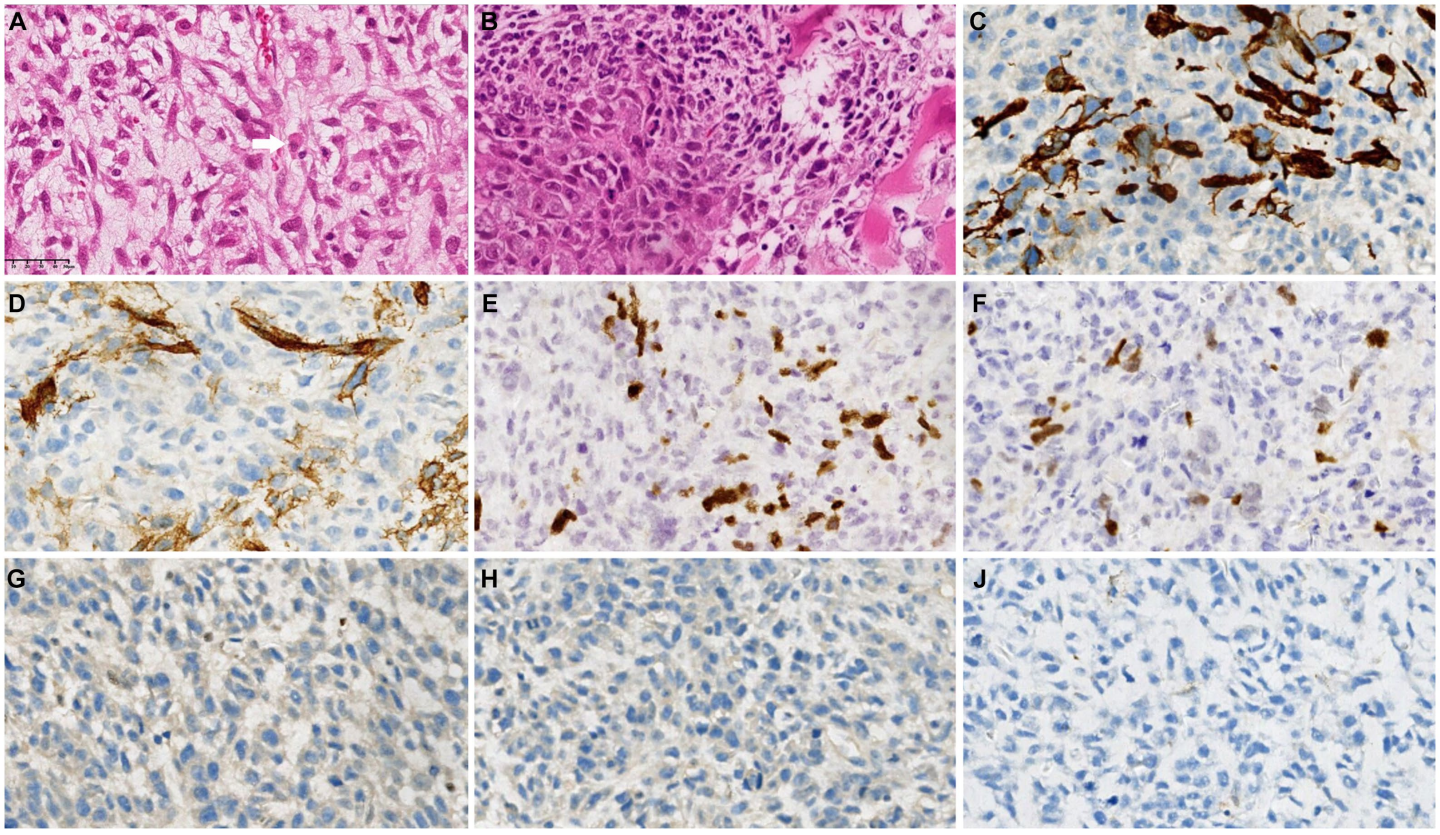
Postoperative histopathological examination revealed a high-grade spindle cell tumor with rhabdomyoblasts (white arrow) and focal cartilaginous components accompanied by tumor-like bone tissue **(A,B)**. Immunohistochemical analysis revealed that the fusiform cells were positive for Desmin **(C)**, CD34 **(D)**, MyoD1 **(E)** and myogenin **(F)** but negative for H3K27me3 **(G)**, S100 **(H)** and SMA **(I)**.

Interestingly, histopathological examination revealed a high-grade spindle cell tumor, with rhabdomyoblasts and focal cartilaginous components accompanied by tumor-like bone tissue (Figures 3A,B). Immunohistochemical analysis (Table 1) revealed that the fusiform cells were positive for myogenic markers (MyoD1 and myogenin), CD34, EMA and Desmin, as well as the loss of H3K27me3 (Figures 3C–I). Furthermore, immunohistochemical staining was negative for the S100, SMA, Syn, CgA, SOX10, AFP and SALL4 proteins. The overall features of the lesion supported those of an MPNST with rhabdomyosarcoma differentiation (also known as “MTT”).

**Table 1:** Immunohistochemical analysis of the tumor.

Immunohistochemical detection	Result
H3K27me3	Negative
Arginase-1	Negative
HepPar-1	Negative
SMA	Negative
S-100	Negative
CK18	Negative
CD117	Negative
TFE3	Negative
Syn	Negative
CgA	Negative
SOX10	Negative
pan-TRK	Negative
AFP	Negative
SALL4	Negative
STAT6	Negative
DOG1	Negative
Calponin	Negative
WT1	Negative
CD34	Positive
INI-1	Positive
EMA	Positive
CD99	Positive
Desmin	Positive
MyoD1	Positive
Myogenin	Positive
TLE1	Positive, nuclear expression
Beta-catenin	Positive, nuclear expression
GS	patchy weak positive
FLI1	Positive
CK (AE1/AE3)	faint expression in a small number of cells

Due to the effect of neoadjuvant therapy, the patient received single TACE and 6 months of lenvatinib plus PD-1 inhibitors after surgery. The patient was followed up at the hospital with no signs of recurrence for 30 months after hepatectomy.

## Discussion

MTT is the most common subgroup of MPNSTs; it is characterized by the presence of rhabdomyoblasts and follows a particularly aggressive course. Most cases of MTTs occur in association with NF1 and are usually observed in people younger than 35 years, without a sex difference (8, 9). The histopathogenesis of this disease has not been determined; however, it is also possible for MTT to develop after radiation therapy. Heads and necks were the most commonly affected areas, followed by the upper and lower extremities as well as the retroperitoneum, buttocks, and trunks. To date, only 18 cases of hepatic MPNST or MTT (primary or metastatic) have been reported in the literature, with only six including a detailed clinical course (10–15) (Table 2).

**Table 2:** Hepatic MPNSTs and MTTs.

Ref	Sex	Age	Primary or metastasis	NF-1	Diagnosis	Hepatic tumor number/size	Treatment for hepatic tumor	Follow-up	Outcome
10	M	64	Metastasis from duodenum	NA	MPNST	1/4.2 cm	Hepatectomy	6 m disease free	alive
11	M	43	Metastasis from retroperitoneal area	No	MPNST	1/1 cm	Hepatectomy	4 m disease free	alive
12	F	72	Primary	No	MPNST	1/18 cm	supportive care after biopsy	16 m	died of disease
13	F	33	Primary	No	MPNST	1/20.0 cm	Right hepatectomy and partial resection of adherent part to diaphragmatic pleura	36 m disease free	alive
14	F	20	Primary	No	MPNST	multiple	Only biopsy	lost to follow-up	NA
15	F	62	Metastasis from duodenum	No	MTT	1/5.5 cm	Radiation, hepatectomy	17 m disease free	alive
Our case	M	56	Primary	No	MTT	1/13 cm	Hepatectomy after neoadjuvant therapy	30 m disease free	alive

MTT, malignant Triton tumor; MPNST, malignant peripheral nerve sheath tumor; NF-1, neurofibromatosis type 1; M, male; F, female; NA, not available.

Histological and immunohistochemical features are important in determining the diagnosis of MTT. Histologically, MTTs exhibit spindle-shaped cells together with large pleomorphic rhabdomyoblastic cells and a wide variety of mitotic figures. Based on the results of immunohistochemistry (IHC) of MTT, spindle cell rhabdomyosarcomas exhibit diffuse expression of desmin, myogenin, EMA, S-100 protein and CD34, as well as the loss of H3K27me3 (16, 17). The loss of H3K27me3 is a sensitive marker for MTT, particularly sporadic MTT, with H3K27me3 being negative in 95% of cases. However, Ohashi K et al. reported a case of metastatic liver MTT with positive immunostaining of H3K27me3 expression, but was lost in the primary duodenal lesion (15). The intratumoral heterogeneity of MTT as a malignant tumor may explain the increased expression of H3K27me3 in metastatic lesions, indicating the presence of tumor cells with intact H3K27me3 in the respective parts of the primary lesion (15). Human cancer is a multidimensional spatiotemporal “ecological and evolutionary unity” pathological ecosystem. Many cancers, if not all, a small subpopulation of cells in tumors termed as cancer stem cells (CSCs) should be the representative for elucidating this evolutionary process because of the capacity of maintaining tumor heterogeneity, unlimited proliferation and evading therapeutic predation (18). In our case, we identified typical high-grade spindle cell tumor, with rhabdomyoblasts. Furthermore, immunohistochemical analysis revealed that the fusiform cells were positive for myogenic markers (MyoD1 and myogenin), CD34, EMA and Desmin, as well as the loss of H3K27me3, confirming the diagnosis of MTT.

The differential diagnosis of MTT includes other spindle cell malignant tumors, such as synovial monophase sarcoma, fibrosarcoma, low-grade fibroblast sarcoma, leiomyosarcoma, especially spindle cell rhabdomyosarcoma (19, 20). In spindle cell carcinoma, there is some evidence of epithelial differentiation or junctional components that will stain positive for keratins. However, our patient’s tumor had only faint pancytokeratin staining (faint expression of CK (AE1/AE3) in a small number of cells and negative expression CK18) and demonstrated no evidence of epithelium on H&E. In synovial sarcomas, fibrosarcomas, and low-grade myofibroblastic sarcomas, there will be no visible rhabdomyoblasts and they will not react to skeletal muscle stains like myogenin, MyoD1 or desmin (19).

Clinical or radiological suspicion is very difficult due to the scarcity of these tumors. Based on CT analysis, Li Y et al. summarized the reference clinical characteristics for the diagnosis of MTT: (i) an enormous mass-like shadow; (ii) a well-circumscribed lobulated shape; and (iii) a well-defined mass with hemorrhages, necrosis, cystic changes and calcification, especially within NF-1 patients (21). In our case, the MTT mimicked hepatocellular carcinoma on MRI and CT, where an enhancement pattern was observed as fast-in and fast-out. Additionally, a history of HBV and a high level of AFP supported the diagnosis of HCC.

In terms of treatment, MTT is similar to MPNST and involves complete surgical resection, whenever possible (11). In addition, adjuvant radiotherapy plays a crucial role in enhancing local disease control. The benefits of adjuvant chemotherapy are unclear, but those who respond to neoadjuvant chemotherapy should also be treated with adjuvant chemotherapy. In the current situation, multimodal management seems to be the best option. The outcome of MTT is generally poor, with only 14% 5-year overall survival (22). The recurrence of MTT after excision is common and greater than that in MPNSTs. In general, MTT has a poor prognosis after recurrence, with an overall survival of 0–18 months after detection (7). In our patient, complete surgical resection of the tumor was performed after 3 months of neoadjuvant therapy (TACE combined with lenvatinib and pembrolizumab). Therefore, the patient received single TACE and 6 months of lenvatinib plus PD-1 inhibitors after surgery. The patient was followed up at the hospital with no signs of recurrence for 30 months after hepatectomy.

## Conclusion

In this report, we describe a rare case of primary hepatic MTT. Although very rare, hepatic MTT is a particularly aggressive tumor characterized by malignant schwannoma with rhabdomyosarcoma and is often associated with NF-1. As a result of the high probability of recurrence and distant metastases, the prognosis of MTT is poor. For hepatic MTT, multifaceted treatment (surgical resection, TACE, pembrolizumab or levatinib) might be the effective therapy.

## Data availability statement

The original contributions presented in the study are included in the article/supplementary material, further inquiries can be directed to the corresponding author.

## Ethics statement

The studies involving humans were approved by Ethics Committee of the Second Affiliated Hospital of Zhejiang University School of Medicine. The studies were conducted in accordance with the local legislation and institutional requirements. The participants provided their written informed consent to participate in this study. Written informed consent was obtained from the individual(s) for the publication of any potentially identifiable images or data included in this article.

## Author contributions

BZ: Conceptualization, Data curation, Funding acquisition, Writing – original draft, Writing – review & editing. CZ: Data curation, Formal analysis, Methodology, Writing – review & editing. YT: Formal analysis, Investigation, Validation, Writing – review & editing. ZG: Data curation, Formal analysis, Investigation, Writing – review & editing. SY: Investigation, Validation, Visualization, Writing – review & editing.
